# Psychological distress among carers and the moderating effects of social support

**DOI:** 10.1186/s12888-020-02571-7

**Published:** 2020-04-06

**Authors:** Emma S. George, Milica Kecmanovic, Tanya Meade, Gregory S. Kolt

**Affiliations:** 1 School of Health Sciences, Western Sydney University, Sydney, Australia; 2 School of Psychology, Western Sydney University, Sydney, Australia

**Keywords:** Psychological distress, Carers, Social support

## Abstract

**Background:**

Carers provide both practical and emotional support and often play an important role in coordination of care for recipients. The demands of caring may lead to increased levels of stress for the carer, which can affect mental health and quality of life. This study examined the relationship between being a carer and psychological distress (assessed using the Kessler Psychological Distress Scale [K10]), and explored the moderating effect of social support in that relationship using a large sample.

**Methods:**

The study used data from the 45 and Up study, a large cohort study of individuals aged 45 years and over in New South Wales, Australia, and applied multiple regression methods and moderation analysis. The sample for the current study comprised 267,041 participants drawn from the baseline dataset, with valid data on the primary outcome (carer status).

**Results:**

The mean age of participants was 62.73 (±11.18) years, and 4.23% and 7.13% were identified as full-time and part-time carers, respectively. Compared to non-carers, full-time carers had K10 scores that were on average, higher by 1.87, while part-time carers’ K10 scores were on average higher by 1.60 points. A perception of social support reduced the strength of the relationship between carer status and psychological distress by 40% for full-time carers and 60% for part-time carers.

**Conclusions:**

The findings have important implications, for both prevention and treatment of psychological problems among carers. In terms of prevention, they suggest that public health campaigns focused on increasing awareness regarding the psychological burden faced by carers would be useful. In terms of intervention, potential treatments that focus on improving social support networks may be helpful. The results are particularly important in the current context of an ageing population in Australian and other developed countries, where caregiving is likely to play an increasing role in the care and support services.

## Background

Almost 2.7 million Australians, or 11.4% of the population, were identified as carers in 2015, and around 856,100 individuals (3.7% of the population) were identified as primary carers [1]. As the percentage of older Australians in the population increases, these figures are likely to grow. Carers provide both practical and emotional support [2] and often play an important role in navigating the available services and coordination of care for the care recipients [3].

### Mental health of carers

The demands of caring can contribute to an increase in the level of stress for the carers, and can affect their quality of life overall, including their mental health [4]. Approximately one-third to one-half of carers have been estimated to experience significant psychological distress, and they experience mental health problems more often than the general population [4]. The level of stress associated with caregiving varies, and this can be influenced by the level of care required, the physical or cognitive impairment experienced by the care recipient, or the duration of the caring relationship [5, 6]. Psychological wellbeing has important implications not only for the carer, but also for the caregiving process as a whole. For example, Pristavec [7] noted that receiving care from caregivers who perceive high burden or few benefits associated with caring can place additional stress on care recipients, negatively impacting their health. Psychosocial variables such as feeling burdened by the carer role, poorer physical and psychological health, and a poorer relationship with the care recipient, have been shown to be associated with an increased risk of institutionalisation of the care recipients with dementia [8].

A meta-analysis of differences between carers and non-carers across caregiving contexts has shown that carers reported being more stressed, depressed, and had lower levels of subjective wellbeing compared to non-carers [6]. For example, the prevalence of high levels of burden among informal carers of people with dementia in the Netherlands was found to be 20%, while a further 58% were found to be at risk of emotional distress as a result of caregiving [9]. In a systematic review, Anastasiadou, Medina-Pradas, Sepulveda and Treasure [10] reported higher anxiety scores for relatives who care for family members with eating disorders compared to those of relatives of healthy family members. Similarly, Barker, Greenberg, Seltzer and Almeida [11] reported that parents who cared for adult children with severe mental illness experienced chronic stress as indicated by physiological markers such as elevated cortisol profiles.

Therefore, the association between caregiving and psychological distress has been established across different caregiving contexts and patient conditions. However, there is also considerable heterogeneity in the psychological distress experienced by carers, and various factors have been shown to influence the psychological distress of carers [12]. Some of those factors include the relationship between the carer and care recipient [13], financial expenditure resulting from the caregiving situation [14], hours of care per day, and complexity of care [15]. Other factors are related to the individual who is being cared for, such as severity of their neuropsychiatric symptoms [16], patients’ level of empathy [17], and frequency of disturbing behaviours [18]. Further, a number of carer-related variables have been found to influence the relationship between caregiving and psychological distress, including gender, resilience [12], sense of competence [14], coping style [19], carer anxiety, household income, and living with the care recipient [20]. Finally, the important role of social support in the carer stress process has also been recognised [21].

While evidence on caregiver burden is well established, it is also important to note that caregiving can provide a sense of meaning and accomplishment. When carers are able to identify and derive meaning from the caregiving role, this can benefit both the carer and the care recipient. In a review of caregiving for cancer patients [22], family caregivers reported improved quality of the relationship with the care recipient, increased feelings of accomplishment, personal strength, patience, and satisfaction relating to their role as a carer. Also identified as a positive aspect of caregiving, was the ability to find meaning within the role, which involved maintaining normality in family life and reprioritising values. In a systematic review on the positive experiences of caregiving in stroke populations, Mackenzie and Greenwood [23] found that carers developed new problem-solving and coping skills that led to increased self-esteem and a sense of mastery. Witnessing small improvements in the care recipient’s health or recovery also led to a sense of pride and satisfaction.

### Social support for carers

Social support may be a protective factor for carers but it depends on type, quality, timing, and duration. Social support is usually divided into two categories: perceived and received social support, where perceived social support is defined as a person’s appraisal of the available social support; and received social support, which is defined as the actual and substantial social support [15]. Social support may involve emotional, tangible, and informational support aspects [21] and can be provided both informally, by family, friends, neighbours, and social groups, or formally by professionals and agencies [24]. It has been asserted that the perceived availability of social support is more important to carers than the actual amount of help received [21], and that it is the quality rather than quantity of social support that is most important [24].

Clyburn et al. [18] investigated the role of received social support in predicting carers’ burden and depression scores in a large sample of carers to people with dementia, in both community and institutional settings. In their study, social support was measured as the number of minutes per week of outside help that the carer received in caring for the patient, further divided into help from informal sources, such as family and friends and from formal services. Receiving little help from family and friends (i.e., help from informal sources) was found to significantly predict carer burden, which in turn predicted higher depression scores among carers. The measure of social support used in the study, however, did not account for how satisfied the carers were with the support that they receive, which may also be an important aspect of social support in the caregiving context.

Social support has also been explored in the context of family functioning, where one or more of the family members are carers. Dimitropoulos, Carter, Schachter and Woodside [25] found that the level of social support reported by carers of individuals with Anorexia Nervosa significantly predicted family functioning, over and above the primary stressor of burden and secondary stressors such as conflict regarding the seriousness of the eating disorder and family conflict regarding attitudes and action toward the person with the eating disorder. The authors postulated that social support may have a positive effect through easing the demands on the family. A lack of social support, on the other hand, may prompt carers to rely more heavily on immediate family, which in turn may negatively impact on family functioning and family relationships. Chiou et al. [15] assessed the role of social support as a predictor of carer burden in a Taiwanese sample of 301 carers to highly dependent family members with disparate diagnoses. They found that perceived social support, but not the number of people in their social support network, was significantly negatively correlated with carer burden.

The relationship between caregiving and psychological distress has been established in the literature, and social support has been identified as an important factor influencing carer stress. Some inconsistencies, however, have arisen regarding the way in which social support acts to reduce carer stress and no previous studies have specifically tested the moderating effect of social support in the relationship between caregiving and psychological distress. Moreover, most of the earlier studies have relied on relatively small and homogenous samples of carers who were providing care to individuals with a specific condition and similar needs. Thus, the carer-stress process identified in the research to date is likely to be highly specific to carers of people with a particular condition, and not generalisable across different conditions and carer contexts.

## Methods

### Aim and design

The aim of this study was to explore the association between caregiving and psychological distress among participants in the 45 and Up study, a large study of healthy ageing in Australia, and to investigate the potential moderating effect of perceived social support on this relationship. It was hypothesised that: 1) carer status is positively associated with psychological distress; and 2) the perceived availability of social support moderates this relationship, so that the effect of being a carer on psychological distress is different depending on the perceived availability of social support.

### Procedure

The data used in this study were drawn from the baseline data of The Sax Institute’s 45 and Up Study, a large cohort study of individuals aged 45 years and over in New South Wales (NSW), the most populous state in Australia. The methods used are described in more details elsewhere [26], but briefly, participants were randomly sampled from the Department of Human Services enrolment database. A total of 267,153 individuals were recruited in the period between January 2006 and December 2009, representing approximately 11% of the adult population aged 45 and over living in NSW. Participants completed a self-report questionnaire, and returned complete documentation via a reply-paid envelope. Participants were required to provide written informed consent in order to participate in the study.

### Participants

Participants for this study included 267,041 participants drawn from the baseline dataset of the 45 and Up Study with valid data on carer status. Individuals who provided care for family or friends were identified based on their response to the question “Do you regularly care for a sick or disabled family member or friend?” Of the 267,041 participants, 30,331 were identified as carers. Descriptive statistics are presented in Table 1.

**Table 1:** Characteristics of the sample with valid data on carer status

Variable	Mean (SD) /Percent	N
**Age in years** (SD)	62.73 (11.18)	267,041
**Female** (%)	53.60%	143,147
**Partnered** (%)	74.69%	199,450
**Health Status**		
Poor	2.12%	5663
Fair	11.58%	30,926
Good	32.59%	87,017
Very Good	35.61%	95,090
Excellent	14.57%	38,915
**Income**		
Less than $5000	1.57%	4185
$5000 - $9999	4.03%	10,749
$10,000 - $19,999	14.09%	37,614
$20,000 - $29,999	9.58%	25,581
$30,000 - $39,999	7.91%	21,111
$40,000 - $49,999	7.22%	19,270
$50,000 - $69,999	10.43%	27,864
$70,000 or more	23.52%	62,807
**Perceived Social Support (%)**	89.56%	239,168
**Carer Status**		
Full-time Carer Status (%)	4.23%	11,283
Part-time Carer Status (%)	7.13%	19,048
**K10 scores**		
Low/no psychological distress (10–15)	65.68%	175,383
Moderate psychological distress (16–21)	13.34%	35,617
High psychological distress (22–29)	4.43%	11,840
Very high psychological distress (30–50)	1.75%	4663

Notes. Partnered, binary variable created to indicate being in a relationship; K10, Kessler-10 Psychological Distress Scale

### Measures

#### Carer status

Participants were asked to report whether they regularly provided care for a sick or disabled family member or friend, and those who responded ‘yes’ were further asked about the time they usually spend caring for this person. Individuals who responded that they cared “full-time” were identified as full-time carers. Those who did not identify themselves as full-time carers were asked to provide the usual hours per week that they spent providing care. These individuals are those who provide care on a regular basis but do not consider themselves to be full-time carers, or do not provide care on a full-time basis; they were identified as part-time carers.

#### Social support

Social support was assessed using the question “How many people outside your home, but within one hour of travel, do you feel you can depend on or feel very close to?” (Duke Social Support Index (DSSI); Landerman, George, Campbell and Blazer [27]). This variable was dichotomised, so that a response of zero was defined as “no social support” and coded 0, and responses of 1 or more were defined as a presence of social support and coded 1. The variable was dichotomised because prior studies [15] have found that the number of people in one’s network does not play a significant role in the psychological distress of carers. Moreover, there is no research suggesting that the relationship between the number of people a person feels they can rely on and psychological distress is linear.

#### Psychological distress

Psychological distress was measured using the Kessler Psychological Distress Scale (K10; Kessler and Mroczek [28]), which is a 10-item questionnaire intended to measure global distress based on questions related to anxiety and depressive symptoms experienced in the preceding 4 weeks. Responses to the 10 items were summed to obtain the K10 scale score, which ranges from 10 to 50. The total scores were categorised into low or no psychological distress (scores of 10–15), moderate psychological distress (scores of 16–21), high (22–29) or very high psychological distress (30–50). The K10 scale has good psychometric properties. For example, using Australian samples, Andrews and Slade [29] found a strong association between high K10 scores and a current diagnosis of a mental disorder, while Slade, Grove and Burgess [30] reported that the K10 is a moderately reliably instrument, with kappa scores that ranged from 0.42 to 0.74.

#### Age

Information on age was available in the 45 and Up Study, based on the reported date of birth at the date of baseline survey completion.

#### Gender

A dichotomous variable for gender was created, coded 0 for males and 1 for females.

#### Marital status

A dichotomous variable (Partnered) was created to indicate whether a person was in a relationship, using information from the question “What best describes your current situation?”, and the response options of single, married, de facto/living with a partner, widowed, divorced, or separated. Individuals who were married or living with a partner/in a de facto relationship were coded 1 and those who reported that they were single, widowed, divorced, or separated were coded 0.

#### Health status

Health status was measured using the question “In general, how would you rate your overall health?” The available responses ranged from 1 (Excellent) to 5 (Poor) and were reverse-coded so that a higher number represented better health.

#### Income

Income was measured using the question “What is your usual yearly household income before tax, from all sources? (please include benefits, pensions, superannuation, etc.)” The available responses were the following yearly income brackets: “less than $5,000;” “$5,000 to $9,999;” “$10,000 to $19,999;” “$20,000 to $29,999;” “$30,000 to $39,999;” “$40,000 to $49,999;” “$50,000 to $69,999;” “$70,000 or more,” and “I would rather not answer this question.”

### Statistical analysis

To examine the association between carer status and psychological distress, a multiple regression was conducted, with K10 as the dependent variable and carer status and social support as the independent variables. The control variables included in the regression were age, gender, being partnered, health status, and income. To estimate the moderating effect of social support on the relationship between carer status and K10 scores, social support was interacted with carer status and the interaction terms were included in the multiple regression analysis. As there were two different carer statuses, full-time carer and part-time carer, two interaction terms were created: full-time carer*social support and part-time carer*social support in order to estimate the moderating effect of social support on (a) the relationship between being a full-time carer and K10, and (b) the relationship between being a part-time carer and K10. Analyses were conducted using STATA version 12 [31].

## Results

### Descriptive statistics

Descriptive statistics for the sample are presented in Table 1. The mean age of the participants was 62.73 (*SD* = 11.18) years, just over a half (52.6%) were female, and most (74.7%) were in a relationship. A total of 4.23% of the sample were full-time carers, and 7.13% were part-time carers.

### Moderation analysis

Table 2 presents the results of the multiple regression analysis, including the moderation analysis, using robust standard errors. The independent variables together explained 18% of the variance in the K10 scores, moderate effect (*R*^*2*^ = .18, *F*(10,179,898) = 2305.47, *ρ* < .001). As can be seen in Table 2, the explanatory variables all individually significantly predicted the dependent variable, K10 scores. Compared to non-carers, full-time carers had K10 scores that were on average higher by 1.87, while part-time carers’ K10 scores were on average higher by 1.60 points. Having social support was associated with lower K10 scores, by 1.55 on average. Females had, on average, higher K10 scores of 0.39. Being older was associated with lower K10 scores, and a one-year increase in age was associated with a K10 score that is lower by 0.09. Being married or having a partner was also associated with lower K10 scores, by 0.80 on average. Moreover, those who reported better general health also had lower K10 scores. Specifically, there was an incremental reduction in K10 scores of 1.91 for each change in the health score (i.e., poor to fair, fair to good, good to very good, very good to excellent). Similarly, there was an incremental change in K10 score of 0.15 for each change in the income bracket.

**Table 2:** Regression of Carer Status, Social Support, and Control Variables on K10 Scores

Variable	*b*	Robust SE	*t*	*p*
Constant	29.06***	0.13	215.72	.00
Age	−0.09***	0.00	−79.93	.00
Sex				
Male (reference)				
Female	0.39***	0.02	17.86	.00
Partner status				
Not partnered (reference)				
Partnered	−0.80***	0.03	−27.42	.00
Health Status	−1.91***	0 .01	− 132.90	.00
Income	−0.15***	0 .01	−24.98	.00
Social Support				
No social support (reference)				
Social Support	−1.55***	0 .06	−25.23	.00
Carer Status				
Non-Carer (reference)				
Full-time Carer	1.87***	0 .23	8.28	.00
Part-time Carer	1.60***	0 .22	7.21	.00
Social Support*Full-time Carer	−0.80***	0 .24	−3.40	.00
Social Support*Part-time Carer	−1.02***	0 .22	−4.59	.00

***p < .001

Both interaction terms (full-time carer*social support and part-time carer*social support) were also statistically significant. The coefficients on both interaction terms were negative, which implies that the effect of social support on the relationship between carer status and K10 scores was negative. As the coefficients on both full-time and part-time carer status variables were positive, the interaction terms revealed that the presence of social support reduces the positive relationship between carer status and K10 scores. Specifically, in the presence of social support, the coefficient on full-time carers is reduced by 0.80, implying that full-time carers with social support have K10 scores lower by, on average, 1.07 points compared to non-carers. In the absence of social support full-time carers have Kessler 10 scores lower by 1.87 on average. Similarly, when social support is included the coefficient for part-time carers is reduced by 1.02 meaning that part-time carers with social support have on average 0.58 lower K10 scores compared to non-carers. On the other hand, part-time carers who report no social support have on average 1.60 points lower K10 scores compared to non-carers.

**Fig. 1:**
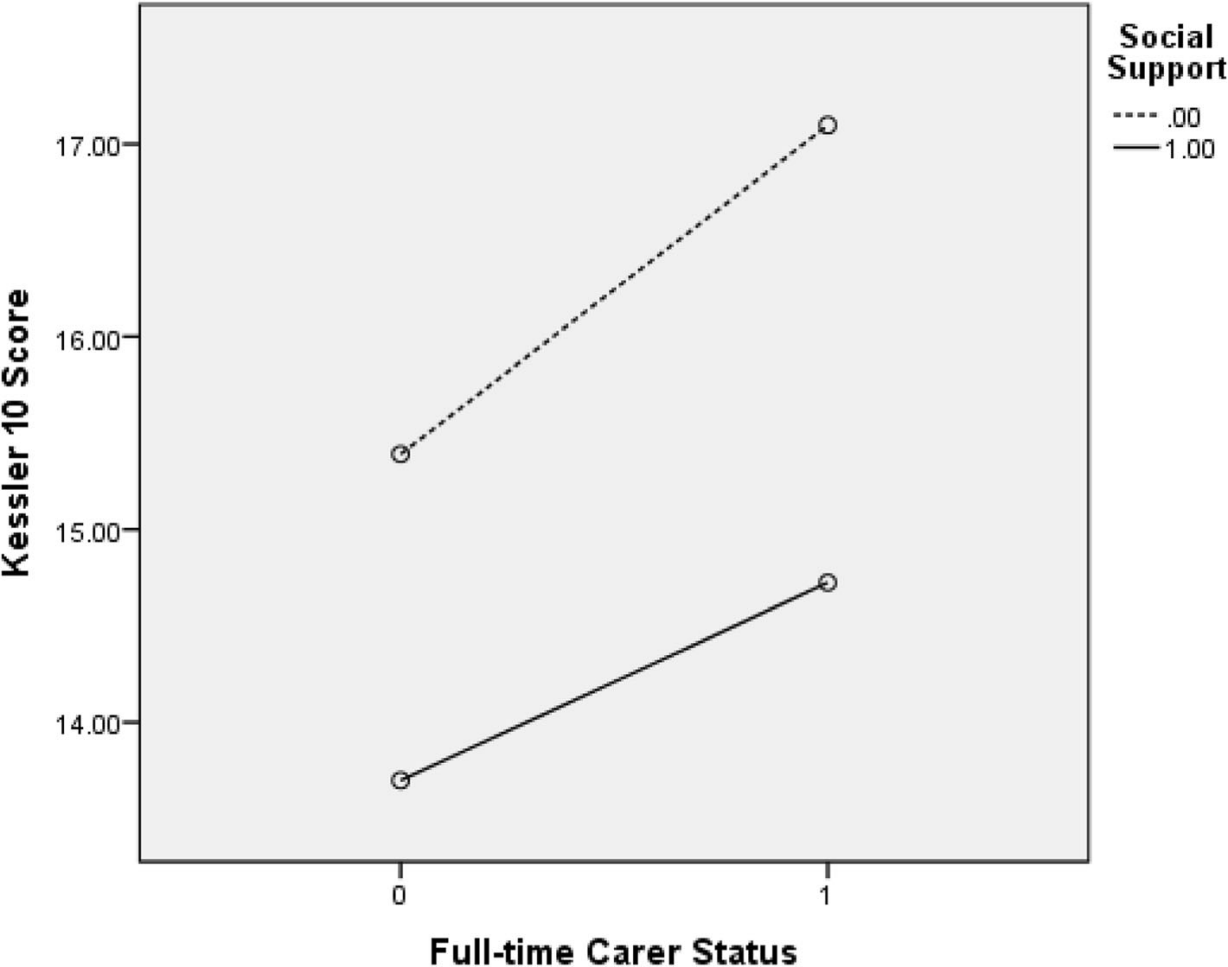
Estimated marginal means of K10 scores and full-time carer status, by social support status

**Fig. 2:**
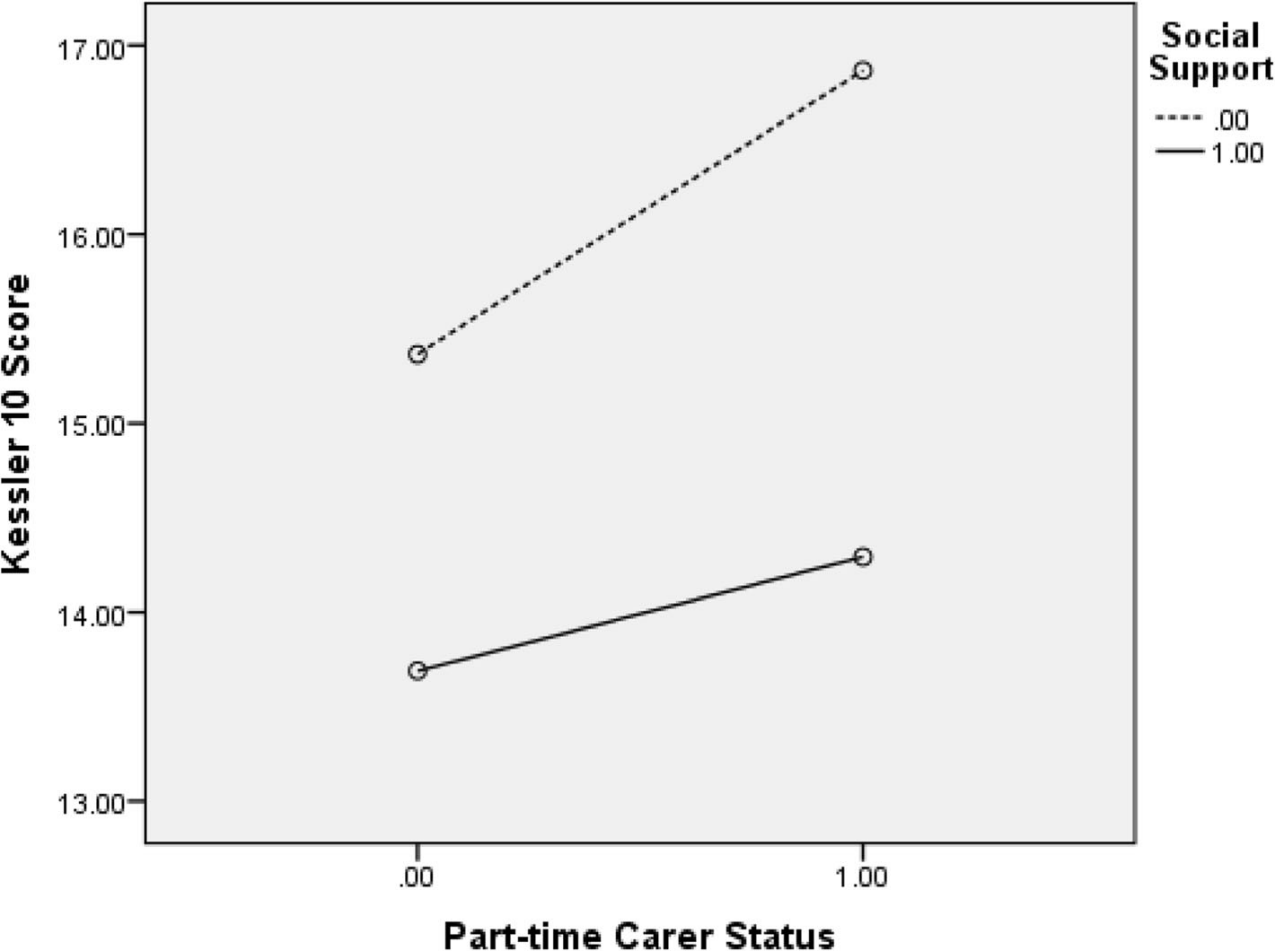
Estimated marginal means of K10 scores and part-time carer status, by social support status

Fig. 1 shows the plot of estimated marginal means of K10 scores and full-time carer status, by social support status. As can be seen in Fig. 1, the line for no social support is steeper than the line for when social support is present. Thus, the relationship between full-time carer status and K10 scores is larger when there is no social support compared to when there is social support. Similarly, Fig. 2 presents the plot of estimated marginal means of Kessler 10 scores and part-time carer status, by social support status. Again, these findings suggest that the relationship between part-time carer status and K10 scores is more impactful when there is no social support, compared to when there is social support.

## Discussion

This study investigated the relationship between carer status and psychological distress, and the moderating effect of perceived social support in that relationship. Both hypotheses were supported: (1) carer status was significantly positively associated with psychological distress for both full-time and part-time carers; and (2) the perceived availability of social support significantly moderated the relationship between carer status and psychological distress, so that the effect of being a carer on psychological distress was significantly smaller when there was perceived availability of social support. The estimated significant relationship between caregiving and psychological distress was small in magnitude, as carer status was associated with a small increase in psychological distress. The moderating effect of social support was, however, large in magnitude; the effect of carer status on psychological distress was around 40% smaller for full-time carers and 60% smaller for part-time carers when there was perceived social support.

The results of this study are consistent with those of previous literature, and extend past findings in three important ways. First, the relationship between carer status and psychological distress has been demonstrated in a number of studies and various caregiving contexts [6, 10]. Most studies that have investigated the psychological distress of carers have relied on small samples of carers within contexts of specific conditions and needs, making it difficult to generalise the results to the wider population. In this study, however, the relationship was confirmed in a large, randomly selected sample of individuals over 45 years of age living in the state of NSW, Australia, and across different care contexts and conditions. Thus, the estimated significant relationship between caregiving and psychological distress, although small in magnitude, may be viewed as more generalisable, given the diverse sample. Furthermore, the important role of social support in the carer stress process has been recognised and is consistent with the findings of several other studies that have shown social support to significantly predict psychological outcomes among carers [15, 18, 25].

Second, while perceived social support has been shown as a significant predictor of psychological functioning of carers, the finding in the present study that perceived social support significantly moderated the relationship between carer status and psychological distress, for both full-time and part-time carers, is novel. Third, although the change in psychological distress scores due to carer status were relatively small in magnitude, the changes in the effect of carer status on psychological distress due to social support were found to be relatively large in magnitude. Specifically, the effect of carer status on psychological distress were about 40% smaller for full-time carers and 60% smaller for part-time carers when there is perceived social support. The magnitude of these interaction effects confirms that perceived social support is indeed an important factor in the stress process of carers.

Understanding the psychological stress associated with being a carer and elucidating the factors that contribute to this stress have important clinical implications, for both prevention and treatment. Although cross-sectional in nature, this study has shown that carers are at an increased risk of experiencing psychological distress, and this is in line with previous findings. To address this, programs could offer greater access to psycho-education and support to carers, and wider campaigns could focus on increasing awareness of the psychological burden that carers face. Although support exists within healthcare systems (e.g., peer support services or the provision of respite services in the community), utilisation of these services is often low or delayed [32, 33]. Additionally, the implementation of such services can be difficult due to resourcing and staffing constraints within existing healthcare services. It may therefore be beneficial to identify opportunities to engage carers through other existing healthcare channels or relationships. McMillan, King, Stapleton, Sav, Kelly and Wheeler [34] for example, delivered a six-step support service for carers through local pharmacies in South Eastern Queensland, Australia. Findings from the program evaluation suggested that this program built staff capacity, enabled a better understanding of carers’ roles, and provided more accessible support for carers.

Moreover, clinicians may see a rise in carers seeking psychological services to assist them through the caregiving process. Thus, the results have important implications for treatment when clients who are carers present to clinicians. In terms of individual therapy, clinicians need to (1) be mindful of the possible psychological consequences on carers when working with them, (2) validate the challenges and psychological stress associated with caring for others, and (3) encourage carers to garner social support, focusing of quality rather than quantity of support.

### Limitations and future research

Several limitations of the study should be considered when interpreting the findings. Firstly, this study used a cross-sectional design that prevents the determination of causality. The 45 and Up Study questionnaire is self-report in nature, and does not provide a clinical confirmation of psychological distress or any additional context relating to the caregiving relationship (e.g., duration of care, tasks undertaken), caregiver population, or quality of social support. Evidence suggests that the level of stress exposure can vary substantially across carer populations (e.g., carers of persons with dementia), and without this contextual information, this cannot be accounted for in the analysis. The dataset utilised for this study was also restricted to individuals aged 45 years or over, so we were not able to include younger carers in our analysis. The study, however, is based on a large-scale survey with a robust analysis and inclusion of reliable scales such as the K10. In terms of extending the current study’s findings, a follow-up longitudinal study of these participants could estimate the moderation effect over time.

## Conclusions

This study has shown in a large, randomly selected sample, that both full-time and part-time carers experience higher psychological distress than non-carers. Moreover, the study has shown that perceived social support significantly moderates the relationship between caregiving and psychological distress, so that the effect of caregiving on psychological distress is reduced for carers with perceived social support, for both full and part-time carers. The findings have important clinical implications in terms of both prevention and treatment of psychological problems among carers. The results are particularly important in the current context of an ageing population in the developed world, where caregiving is likely to grow in magnitude.

## Data Availability

The data that support the findings of this study are available from The Sax Institute, but restrictions apply to the availability of these data, which were used under license for the current study, and so are not publicly available. The dataset used in the analysis of this study is available upon reasonable request and with permission of The Sax Institute.

## Abbreviations

K10: Kessler 10 Psychological Distress Scale
DSSI: Duke Social Support Index
NSW: New South Wales
